# Supplementary material for: An open-source framework for large-scale, flexible evaluation of biomedical text mining systems
Source: J Biomed Discov Collab. 2008 Jan 29;3:1. doi: 10.1186/1747-5333-3-1 (PMC2276192; doi:10.1186/1747-5333-3-1)
Supplement: Additional file 1 — Evaluation platform source code. The additional file contains the software evaluation framework discussed in this paper. The most current release can be downloaded from SourceForge.net [1]. See the accompanying README file for instructions on installation and use. Also included in the distribution is a collection of UIMA wrappers for some commonly used BioNLP tools and annotated corpora. [file 1747-5333-3-1-S1.zip › bionlp_uima_repository-1.0.1/docs/api/edu/uchsc/ccp/util/nlp/document/DocumentSectionTypes.html]

DocumentSectionTypes (Center for Computational Pharmacology Util/UIMA API)


|  |  |  |
| --- | --- | --- |
| **Overview** \| **Package** \| **Class** \| **Tree** \| **Deprecated** \| **Index** \| **Help** | |  |
| **PREV CLASS**   **NEXT CLASS** | **FRAMES**    **NO FRAMES**     **All Classes** |  |
| SUMMARY: NESTED \| FIELD \| CONSTR \| METHOD | DETAIL: FIELD \| CONSTR \| METHOD |  |


---


## edu.uchsc.ccp.util.nlp.document Interface DocumentSectionTypes

---

``` public interface DocumentSectionTypes ```

---

| **Field Summary** | |
| --- | --- |
| `static int` | `ABSTRACT_ID` |
| `static String` | `ABSTRACT_NAME` |
| `static int` | `HW_ABSTRACT` |
| `static int` | `HW_TITLE` |
| `static int` | `OTHER_ID` |
| `static String` | `OTHER_NAME` |
| `static int` | `TITLE_ID` |
| `static String` | `TITLE_NAME` |

**Field Detail**

### OTHER\_NAME

```
static final String OTHER_NAME
```

**See Also:**: Constant Field Values

---


### OTHER\_ID

```
static final int OTHER_ID
```

**See Also:**: Constant Field Values

---


### TITLE\_NAME

```
static final String TITLE_NAME
```

**See Also:**: Constant Field Values

---


### TITLE\_ID

```
static final int TITLE_ID
```

**See Also:**: Constant Field Values

---


### ABSTRACT\_NAME

```
static final String ABSTRACT_NAME
```

**See Also:**: Constant Field Values

---


### ABSTRACT\_ID

```
static final int ABSTRACT_ID
```

**See Also:**: Constant Field Values

---


### HW\_TITLE

```
static final int HW_TITLE
```

**See Also:**: Constant Field Values

---


### HW\_ABSTRACT

```
static final int HW_ABSTRACT
```

**See Also:**: Constant Field Values


---


|  |  |  |
| --- | --- | --- |
| **Overview** \| **Package** \| **Class** \| **Tree** \| **Deprecated** \| **Index** \| **Help** | |  |
| **PREV CLASS**   **NEXT CLASS** | **FRAMES**    **NO FRAMES**     **All Classes** |  |
| SUMMARY: NESTED \| FIELD \| CONSTR \| METHOD | DETAIL: FIELD \| CONSTR \| METHOD |  |


---
